# Autoimmune liver disease and multiple sclerosis: state of the art and future perspectives

**DOI:** 10.1007/s10238-023-01128-8

**Published:** 2023-07-08

**Authors:** Rosanna Villani, Gaetano Serviddio, Carlo Avolio, Tommaso Cassano, Emanuele D’Amico

**Affiliations:** 1 Liver Unit, Department of Medical and Surgical Sciences, University of Foggia, Foggia, Italy; 2 Department of Medical and Surgical Sciences, Multiple Sclerosis Center, University of Foggia, Foggia, Italy; 3 Department of Medical and Surgical Sciences, University of Foggia, Foggia, Italy

**Keywords:** Liver, Autoimmune hepatitis, Primary biliary cholangitis, Multiple sclerosis

## Abstract

Clinical observations suggest that the prevalence of autoimmune diseases is changing over time. Both autoimmune liver diseases and multiple sclerosis have shown a significant increase in the last decades. Although the coexistence of autoimmune diseases within individuals and families is a common phenomenon, the extent to which liver disease and multiple sclerosis co-occur is not clear. Case reports and few studies have reported the possible coexistence of multiple sclerosis with thyroid diseases, inflammatory bowel disease, psoriasis, and rheumatoid arthritis. It is unknown whether there is a definite association between multiple sclerosis and autoimmune liver diseases. We reviewed the literature to summarize the available studies on the association between different autoimmune liver diseases (autoimmune hepatitis, primary biliary cholangitis, and primary sclerosing cholangitis) and treated or untreated multiple sclerosis.

## Introduction

The immune system fulfills the major function of host defense against infectious agents and eliminates endogenous challenges such as abnormal or damaged cells [1, 2].

Normally, it is able to distinguish self from non-self-molecules (antigens), a process known as “immune tolerance” which is the normal state of immune unresponsiveness and inertness to self-molecules, which prevents the targeting of self-cells and self-tissues [2].

The “break of tolerance” is defined as the failure to distinguish self from non-self and is the basis for autoimmune diseases [3].

The concept of autoimmunity was described for the first time by Nobel Laureate Paul Ehrlich. In 1900, he showed that the immune system is focused on responding to non-self molecules and has an intrinsic tendency to avoid attacking self-tissues [4]. He labeled the natural aversion of the immune system in reacting to self-antigens as “horror autotoxicus” [5].

For many decades, this concept was wrongly understood to mean that autoimmunity could not exist and that a self-destructive process could not explain the pathogenesis of systemic diseases [6].

For this reason, scientists avoided using the term “autoimmunity” until the middle of the twentieth century. At that time, autoimmune diseases were considered as concealed forms of allergy, whereas the most severe form of self-induced injury was called anaphylaxis [7].

The discovery in 1956 that Hashimoto thyroiditis described for the first time in 1912 is an autoimmune disease introduced the concept of human autoimmunity [8].

Historically, autoimmune diseases are considered rare; however, rigorous epidemiological studies have shown a general prevalence of 3–5% with thyroid disease and type I diabetes being the most frequently observed autoimmune disease [9, 10].

Autoimmune diseases can occur at any age, but specific features can be described in different subgroups.

The incidence and prevalence of autoimmune diseases differ between geographical areas.

For example, the incidence of multiple sclerosis has been reported to be 0.7–3.6 per 100 000 person-years in Asia and the Middle East versus 2.7–7.5 per 100 000 person-years in North America [2, 11]. Similarly, the incidence of type-1 diabetes is 10–20 and < 1 per 100 000 person-years in populations from US and China, respectively [2].

These data suggest that both genetic susceptibility and gene/environment interaction are key risk factors involved in the loss of tolerance [12].

Several risk factors for autoimmunity, including genetic variants, are shared among various conditions, suggesting that additional autoimmune disorders may be observed in patients with a history of autoimmune disease and, in fact, it is known that 25% of patients with at least one autoimmune disorder can develop additional autoimmune diseases [13, 14].

The prevalence of autoimmune diseases is typically increased in women (female-to-male ratio ranging from 10: 1 to 1: 1), first-degree relatives, and in monozygotic twins, suggesting that genetic susceptibility plays a key role [15] even if the concordance rate in monozygotic twins is between 12 and 67%. These data are consistent with the evidence that environmental factors play a pivotal role in triggering the autoimmune cascade.

Autoimmune liver diseases include three main distinct complex disorders that have well-defined clinical phenotypes, patterns of inflammation, and serologic profiles: autoimmune hepatitis (AIH), primary biliary cholangitis (PBC), and primary sclerosing cholangitis (PSC) [16].

In the general population, the overall incidence of autoimmune liver diseases is 1–2 per 100,000 population per year even if different incidence rates have been observed over time and place suggesting that additional risk factors may promote the development of autoimmune liver disorders [17].

Autoimmune liver disorders are complex diseases resulting from the interaction between genetic and environmental factors. Recent genome-wide association studies (GWAS) and iCHIP-association studies identified several risk loci involved in autoimmune responses in patients with autoimmune diseases [16]. These studies have improved the understanding of the pathophysiology of autoimmune liver disease and explained the higher risk of individuals and their families to have multiple autoimmune conditions [16].

Multiple sclerosis (MS) is a chronic inflammatory disease of the central nervous system that affects more than 2 million people worldwide [18]. Both genetic and environmental risk factors have been linked to MS even if no specific etiological trigger has been identified [19]. Individuals with an affected first-degree relative have 2–4% risk of developing MS (versus about 0.1% in the general population), and concordance in monozygotic twins is 30–50% [20].

Three-quarters of people with MS are women, and for several reasons MS is considered an autoimmune disease [20]. Genome-wide association studies have identified > 200 gene variants that are associated with the risk of MS. Most risk alleles have to deal with immune-regulatory pathways, consistent with the notion that autoimmune mechanisms play a key role in the development of clinical MS [20].

Clustering of autoimmune diseases within individuals and families is a common phenomenon [21]; however, the extent to which MS and other autoimmune diseases co-occur is unknown. Several case reports and larger studies have reported the possible co-occurrence of MS with thyroid diseases, systemic lupus erythematosus, scleroderma, myasthenia gravis, ulcerative colitis, psoriasis, and rheumatoid arthritis. There is less evidence that MS may coexist with autoimmune liver diseases [22].

## Autoimmune hepatitis and multiple sclerosis

### Autoimmune hepatitis: pathogenesis, diagnosis, and clinical presentation

Autoimmune hepatitis (AIH) is a chronic disease that affects mainly women and is characterized by circulating autoantibodies, high levels of gamma globulins, and interface hepatitis on liver histology [23–25].

AIH is considered a rare disease because its prevalence ranges from 15 to 25 cases per 100,000 inhabitants, even if the incidence is increasing over time in both women and men [24].

A large nationwide population-based study in Denmark showed that during a 20-year-period the incidence rate of AIH has nearly doubled [26] and, similarly, in a population-based prospective study in New Zealand, the incidence of AIH was significantly higher in 2014–2016 period than the 2008–2010 period, whereas incidences of PBC and PSC were unchanged over the same period [27].

Moreover, ethnicity seems to affect the AIH prevalence, severity of clinical expression, and mortality; therefore, the prevalence of AIH in Europe is up to 18 cases per 100,000 inhabitants, whereas prevalence rates of 43 cases per 100,000 inhabitants have been reported in Alaskan natives. North American Aboriginal and African American patients have a more severe disease, a higher frequency of treatment failure, and a higher mortality [28, 29].

A number of triggering factors have been proposed, including viruses and drugs, but none has been conclusively shown to cause autoimmune liver diseases.

Recently, Lammer et al. found that environmental factors may promote the pathogenesis of AIH because AIH patients had more urinary tract infections, recurrent urinary tract infections, and a higher vaccination frequency to chicken pox, measles, mumps, rubella pertussis, and pneumococcus. Several authors have also reported the association between hepatitis A, B, C, D, and E viruses or CMV, EBV, HSV-1, and autoimmune hepatitis [30]. Moreover, some drugs such as nitrofurantoin and minocycline have been associated with 90% of drug-induced AIH suggesting that medication may be associated with autoimmune liver diseases [31].

After exposure to environmental triggers, the absence of effective B regulatory cell (Breg) inhibition, inability of natural T regulatory cells (nTregs), and inducible T regulatory cells (iTregs) do not block autoreactivity [32, 33].

Antigen presentation can occur by direct or indirect mechanisms: a. direct presentation: the antigen is presented by DCs (MHC-I molecules) to CD8 + T cells; b. cross-presentation: the uptake of dying cells and the subsequent presentation by MHC-I molecules to CD8 + T cells; c. cross-dressing: MHC-I-peptide transfer from an APC or tumor cell to a DC via trogocytosis or exosomes and then activation of CD8 + T cells without antigen processing by the DCs; d. MHC-II dressing: intercellular MHC-II transfer (via trogocytosis/exosomes) of exogenous antigen-MHC-II complexes from DCs to close DCs, CD4 + T cells, or natural killer (NK). Trogocytosis is defined as the cell–cell contact and T cell receptor (TCR)-dependent membrane transfer of peptide-loaded pMHC-I and pMHC-II complexes between T cells and professional or unconventional APCs [34, 35].

In the liver, both parenchymal and nonparenchymal cells (hepatocytes, cholangiocytes, liver sinusoidal endothelial cells, hepatic stellate cells, and liver-resident leukocytes) are APCs able to perform trogocytosis or produce extracellular vesicles/exosomes [36]. Recently, trogocytosis and intercellular transfer of peptide-loaded human leukocyte antigen (HLA) molecules via extracellular vesicles process have been causally linked to AIH and PBC pathogenesis because they have been shown to promote and amplify immune responses, induce of anergy, and exhaust T effector cells. In particular, trogocytosis has been shown to be involved in “immunological synapse” between the T cell receptor and antigen-presenting MHC-II-expressing hepatocytes, which leads to the acquisition of immune complexes by CD4 + T cells, and finally, repeated hepatocyte injury [35].

It is well known that AIH is a clinically heterogeneous syndrome, including several clinical, laboratory, and histological manifestations that can lead, if untreated, to cirrhosis, liver failure, and death [33].

A predilection for young women and a favorable response to immunosuppression are typical features of AIH. Only 25% of cases are males and a primary non-response to immunosuppressive treatment is experienced in a small number of patients with AIH; therefore, in case of non-response, a careful reevaluation of the diagnosis or adherence to treatment should always be considered [24].

According to the pattern of autoantibodies detected and clinical characteristics, different subclasses of AIH can be observed. Two major types, type 1 and type 2 AIH, have been identified [24].

Type 1 AIH is characterized by the presence of ANA and/or ASMA, whereas type 2 AIH is characterized by the detection of specific anti-liver/kidney microsomal antibody type 1 (anti-LKM1) or less frequently anti-LKM type 3 (anti-LKM3) and/or antibodies against liver cytosol type 1 antigen (anti-LC1) [23, 25, 37, 38].

AIH-1 is characterized by variable clinical and histopathological severity, rare failure of treatment but need for long-term maintenance therapy. AIH-2 develops in childhood and young adulthood, and its clinical severity is generally acute; frequent failure of treatment and relapse after drug withdrawal have been observed.

The majority of adult patients with AIH are affected by AIH-1 (95%). AIH-1 affects people of all ages with two peaks, the first between 10 and 18 years of age and the second one around the age of 40 years [25]. About 20% of AIH-1 patients are diagnosed after the age of 60 years [25, 39].

AIH-2 mainly affects children, including infants (< 1 year of age) and adolescents and young adults (< 25 years of age), whereas it is rarely found in patients aged 25 years or older [25, 39].

ANA are detected in 80% of adults with AIH at presentation, ASMA are present in 63% and anti-LKM1 in 3% of patients [40].

Up to 20% of AIH cases are negative for ANA, ASMA, and LKM1 autoantibodies. In this case, other autoantibodies may be sought, such as Anti-SLA, perinuclear antineutrophil cytoplasmic antibodies (pANCA), or Anti-LC1. Anti-SLA has high specificity (99%) but is present in only 7%-22% of patients with type 1 AIH [33, 41–43]. pANCA are detected in patients with type 1 AIH (50%-92%), but they lack diagnostic specificity being detectable also in primary sclerosing cholangitis, ulcerative colitis (UC), and drug-related liver injury [44, 45].

### Genetic risk factors for autoimmune hepatitis and multiple sclerosis

Although the etiology of autoimmune hepatitis and MS is unknown, genetic and environmental risk factors are thought to be involved in their pathogenesis.

AIH develops in genetically predisposed individuals after exposure to environmental factors, leading to loss of self-tolerance and immune-mediated injury.

Genetic studies have shown that predisposition to developing AIH can be attributed to polymorphisms of the human leukocyte antigen (HLA) region, encoding the major histocompatibility complex (MHC).

The relevant role of genes encoded in the HLA region has been confirmed by large genome-wide association studies that showed in different populations different risk genotypes for AIH. In Europe and North America, susceptibility to AIH-1 in adults is conferred by HLA-DR3 (HLADRB1*0301) and HLA-DR4 (HLADRB1*0401) genotypes [46, 47].

In Japan and South America, susceptibility is associated with HLADRB1*0405 and HLADRB1*0404 alleles [48].

AIH-2 is associated with specific HLA class II susceptibility alleles; DQB1*0201 is considered the main determinant of susceptibility, whereas DRB1*07/DRB1*03 is associated with the type of autoantibody present [25, 49, 50].

In addition to genetic predisposition, the breakdown of self-tolerance mechanisms includes the loss of the homeostatic process based on the control of circulating autoreactive T cells, which can cause tissue damage. The control of the autoreactive T cells is exerted by regulatory T (Treg) cells. Among T cell subsets with potential immunoregulatory function, Treg cells CD4 + T lymphocytes constitute 5–10% of all peripheral CD4 + T cells in healthy individuals and constitutively express the IL-2 receptor subunit-α (IL2-RA; also known as CD25). They control innate and adaptive immune responses by limiting the proliferation and effector function of autoreactive T cells.

A numerical and functional defect in Treg cells has been observed in AIH patients [25]. In AIH patients, the number of circulating Treg cells is lower than in healthy individuals, and this reduction is more evident before treatment and during relapses. The number of Treg cells correlates inversely with Anti-SLA and Anti-LKM1 autoantibody titers, suggesting that a reduction in the number of Treg cells facilitates the manifestation of AIH. Finally, Treg cells derived at diagnosis from patients with AIH have a functional defect with a lower ability to control the proliferation of CD4 + and CD8 + effector cells than Treg cells isolated from healthy individuals [25].

Multiple sclerosis (MS) is an autoinflammatory disease in which the oligodendrocytes are progressively destroyed with subsequent loss of neuronal function [51]. The risk of developing MS significantly increases in case of relatedness to someone who is affected by MS. Genome-wide association studies (GWAS) suggest that variation in the regulatory regions of immune genes is linked to increased susceptibility to MS and more than 200 genes have now been identified as risk genes accounting for approximately half of MS heritability [52, 53].

The largest and first MS risk gene variant is HLA-DRB1*15:01 which increases risk by about threefold and affects pathogenesis more than the other known risk variants [54]. HLA-DRB1 is expressed in antigen-presenting cells because it presents peptides to CD4 T cells and regulates their activation.

Unlike other DRB1 alleles, the structure of the 15:01-binding groove is able to present both myelin and viral peptides (e.g., EBV) to T cells. Therefore, molecular mimicry could contribute to T cell activation through DRB1*15:01 [55].

Additional MHC class I alleles with protective effects have also been identified [53]. They are involved in presenting antigens to CD8 T cells, or interact with natural killer (NK) cells.

CD8 T cells responding to peptides presented by protective alleles may be more effectively activated to kill infected cells or autoreactive T cells [53].

Differentiation of immune cells is different in individuals and is controlled by genetic variants of transcription factors, cytokine receptors, or signaling molecules. TYK2, IL2Ra, EOMES, and NFKB1are MS risk genes associated with immune dysregulation in MS.

Moreover, some authors have demonstrated that the difference in response of immune cell subsets to the cytokines IL-7 and IL-2 could be highly heritable and that these genes and/or their receptors are MS genetic risk factors [53, 56].

IL2RA, also known as CD25, associates with CD122 and forms the high-affinity receptor for IL2. It plays a central role in the balance between immune tolerance and autoimmunity. IL-2 signaling is important for the survival and suppressive capacity of Treg cells and impacts the fate of T cells (memory and effector differentiation) [57].

Scientific literature shows several similarities in the genetic background of AIH and MS. Most literature showed that main determinants of susceptibility, both in AIH and MS, may be specific MHC class alleles such as HLA-DRB1variants, which are involved in presenting peptides to CD4 T cells and regulation of their activation and impaired IL-2/IL-2RA signaling, which is pivotal for the maintenance of the ability of Treg to control autoreactive cells.

### Autoimmune hepatitis in untreated MS

**Fig. 1:**
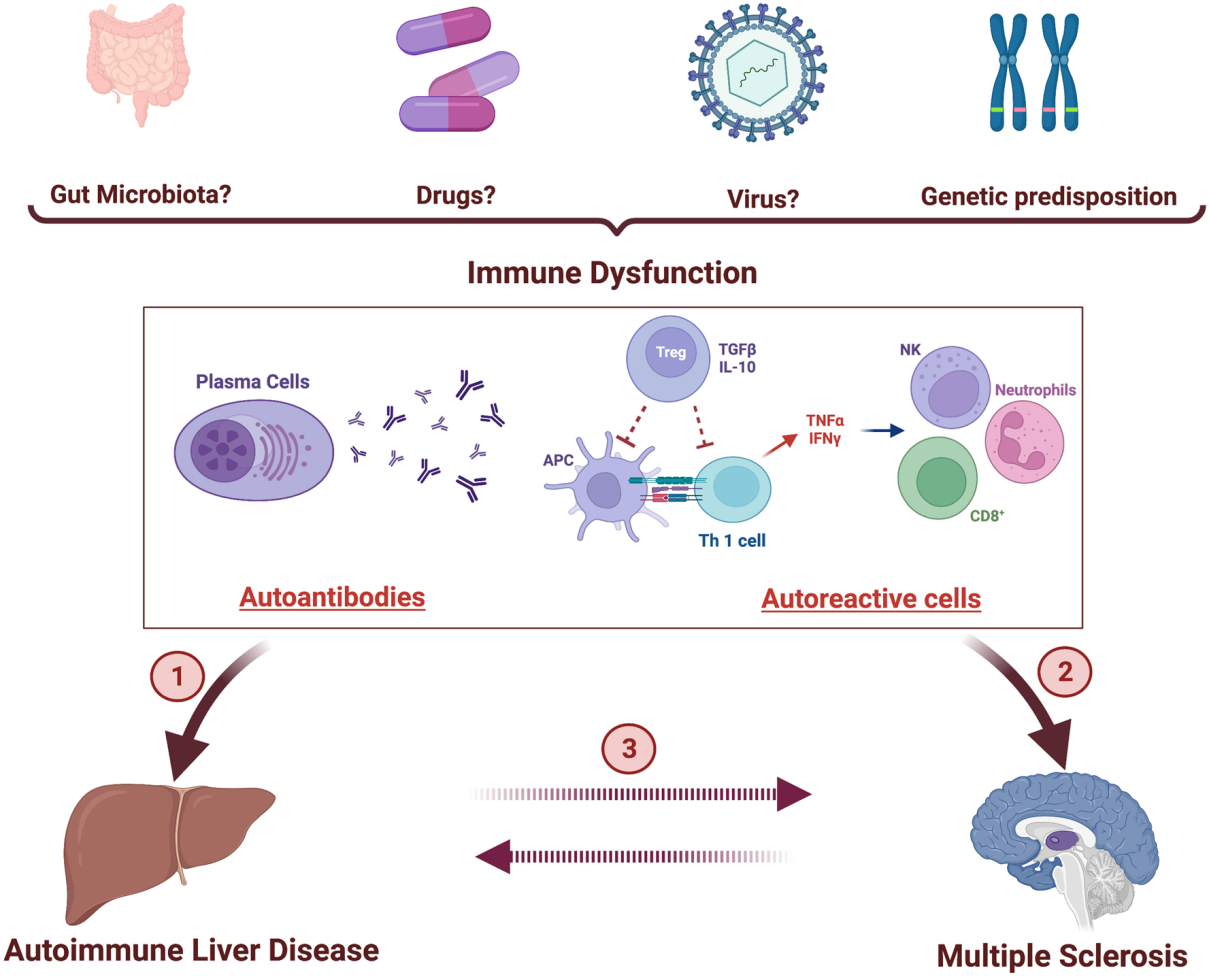
Immune dysfunction due to genetic and environmental factors is a common feature of both autoimmune hepatitis ① and multiple sclerosis ②. The direct association of multiple sclerosis and autoimmune hepatitis has not been confirmed; however, epidemiological data suggest that the occurrence of multiple sclerosis increases the risk of autoimmune hepatitis and vice versa ③. (Picture created with BioRender.com)

AIH has been associated with multiple sclerosis [58, 59], and its prevalence is estimated to be 0.17% versus 0.02% in the general population, which raises the question whether humoral mechanisms may play a significant role in their association [60] (Fig. 1).

There are very few studies addressing the association between autoimmune liver disease and autoimmune CNS disorders.

However, there is strong evidence that abnormal immune responses against self can result in more than 80 autoimmune diseases [61] and that about 30 autoimmune disorders involve the nervous system.

Therefore, a potential coexistence of autoimmune liver and CNS disorders, more than that currently reported in the literature, may be expected.

*Tsouris *et al. assessed the prevalence of autoantibodies for AIH in a cohort of MS patients. The authors enrolled 133 patients (30 naïve MS patients and 103 treated patients) and 26.7% of naïve MS patients had at least one of the autoantibodies vs 21.4% of MS under treatment and 8% of patients included in the control group. ASMA were the most frequent antibody detected (20% in naïve patients vs 11.7% in treated patients). ANA was detected in 8 patients out of 133 and anti-LKM-1 in one patient, whereas no patients had Anti-SLA/LP or Anti-LC1. Among MS patients with at least one autoantibody positivity (*N* = 30) only 2 patients had overt type 1 AIH [62].

In 2015, *Marrie *et al. performed a systematic review of the literature to estimate the incidence and prevalence of autoimmune disease in MS. The most commonly reported autoimmune comorbidities in MS patients were psoriasis (7.74%) and thyroid disease (6.44%). Concerning the incidence and prevalence of autoimmune hepatitis, the authors concluded that no differences were found between the general population and MS patients, even if only two studies were included in the final analysis [63].

Table 1 summarizes the currently available publications reporting cases of coexistence of untreated MS and AIH.

**Table 1:** AIH in untreated MS

Author	Country	Number of patients	Age (years)	Gender	Autoantibodies	Liver biopsy	AIH treatment
Rigopoulou et al. [64]	Greece	1	n.a	n.a	n.a	n.a	n.a
Nadhem et al. [60]	US	1	61	F	ASMA	Yes	Prednisone Azathioprine
Farkas et al. [65]	US	2	61 (patient 1) 56 (patient 2)	F	ANA + ASMA(patient 1) Negative (patient 2)	Yes	Steroids
De Seze et al. [66]	France	3	41 (patient 1) 33 (patient 2) 40 (patient 3)	F (patient 1) M (patient 2) M (patient 3)	Negative	Yes	Azathioprine Steroids
Nunez et al. [67]	Spain	2	25 (patient 1) 28 (patient 2)	F	Negative	Yes	Azathioprine Steroids
Tsouris et al. [62]	Greece	2	n.a	n.a	n.a	n.a	n.a

n.a. not available

Only eleven cases are available, and most patients had a good clinical response to the recommended treatment schedule for AIH.

*Rigopoulou *et al. observed between 2005 and 2017 184 patients with MS; 14 patients also suffered from AIH. Thirteen patients were under treatment, and only 1 patient was untreated. All patients had detectable ASMA (100%), whereas ANA and anti-LKM-1 were detected in 36% and 14%, respectively [64]. Anyway, the authors reported only pooled results, whereas specific data on the untreated patient were not available. *Nadhem *et al*.* reported one case of AIH in a 61-year-old patient who presented with fatigue, right upper quadrant abdominal pain, and increase ALT levels (max 1497 U/L). Serological tests revealed positive antismooth muscle antibodies at 1:320 (normal titer < 1:40), whereas liver biopsy showed lobular lymphoplasmacytic infiltrate, Councilman bodies, and bridging periportal fibrosis consistent with autoimmune hepatitis. The patient had not taken any treatment for MS, except for interferon 3 years before the AIH diagnosis [60].

*Farkas *et al. reported 2 case of AIH in patients with untreated MS. The first one, a 61-year-old woman had autoantibodies (ANA and ASMA) positive, whereas liver biopsy showed panlobular necrosis and plasma cells. The second case, a 56-year-old untreated woman with MS presented with jaundice and fatigue. She had elevated liver enzymes and bilirubin, whereas ANA and ASMA were negative. Liver biopsy showed plasma cells and interface hepatitis consistent with AIH. Both of them responded to steroid therapy [65].

*De Seze *et al. screened 1800 patients with MS every year for liver enzymes. During the seven-year clinical follow-up, the authors observed a significant increase in liver enzyme levels in five untreated patients (0.28%). Autoantibodies were negative in all cases and all patients underwent liver biopsy. AIH was confirmed in three out of five patients (0.17% of the whole cohort), whereas the remaining two patients had steatosis.

After diagnosis, all patients with AIH were treated with azathioprine and corticosteroids and showed a good response to treatment [66].

Finally*, Nunez *et al. reported 2 cases of autoimmune hepatitis; one patient developed fulminant hepatic failure requiring liver transplantation. Serum autoantibodies were negative in both patients, even if the clinical course and liver biopsy showed typical features of AIH [67].

**Fig. 2:**
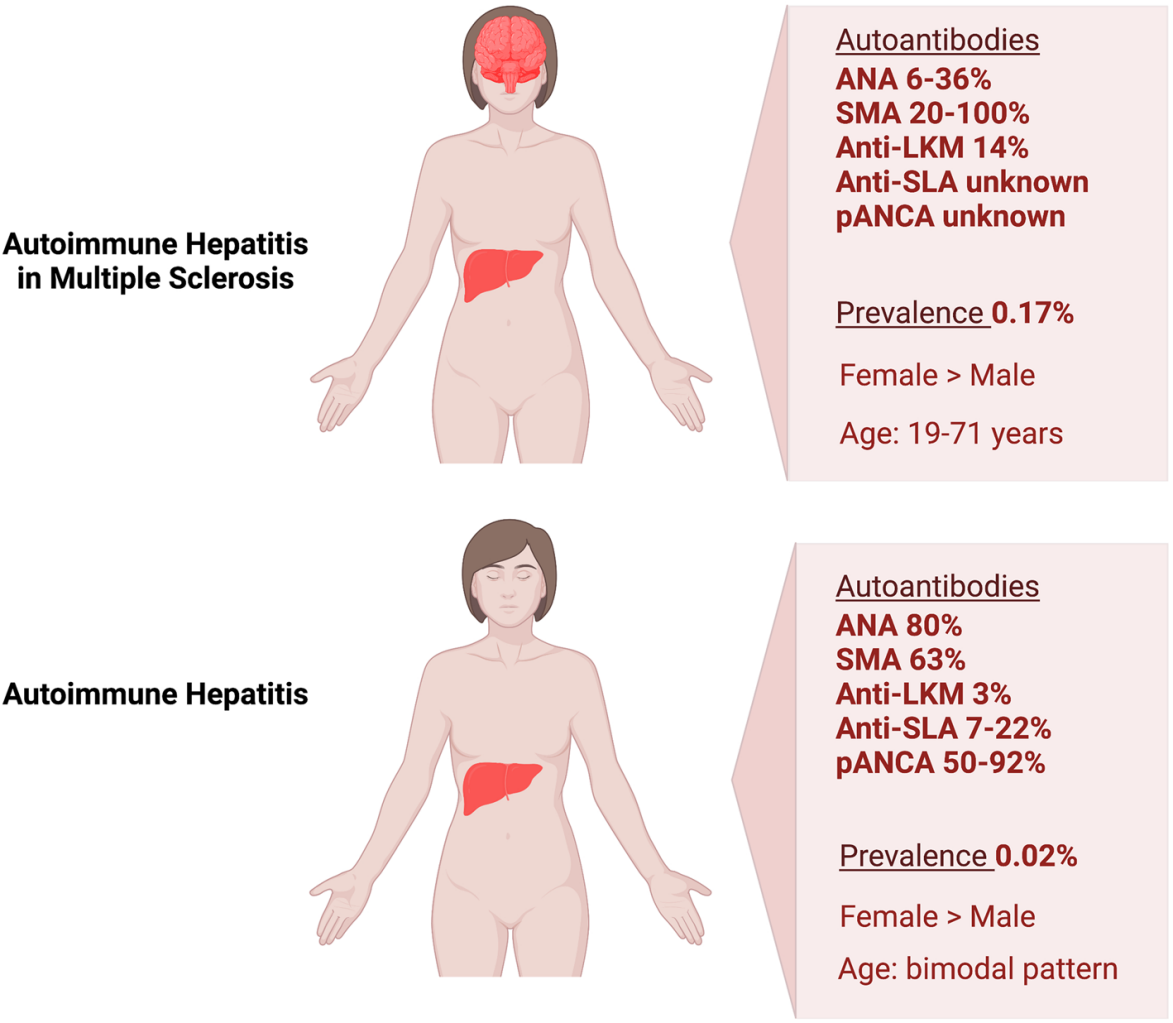
Epidemiology and serum biomarkers of autoimmune hepatitis in patients with multiple sclerosis in comparison with features of autoimmune hepatitis alone. (Picture created with BioRender.com)

The revision of the literature suggests that there are different biochemical and, then, clinical features for AIH in MS patients. The prevalence and type of autoantibodies in patients with AIH and MS were different compared with patients with AIH alone (Fig. 2). A lower prevalence of ANA and a higher prevalence of seronegative AIH have been reported and may be involved in underestimation of AIH. Furthermore, the lower detection of autoantibodies suggests that the occurrence of hypertransaminasemia may be more often misdiagnosed as drug liver injury when liver biopsy is not available, especially in patients taking medication for MS treatment. Liver biopsy remains an essential tool to confirm AIH in all MS patients with alteration of liver enzyme irrespective of detectable autoantibodies and treatment, and currently available guidelines recommend histological assessment of the liver for diagnosis in case of abnormal liver tests of unclear etiology.

### Autoimmune hepatitis in treated MS

Currently, there is no definite cure for multiple sclerosis, and current therapy focuses on speeding recovery from attacks, slowing the progression of the disease, and managing symptoms.

Before 1996, corticosteroids and unselective immunosuppressive drugs (azathioprine, cyclophosphamide, methotrexate) were available for relapses and symptomatic patients with a low evidence of any efficacy [68].

However, the management and treatment of MS has become more complex over the last two decades and after the approval of interferon beta-1b (IFNβ) as the first disease-modifying drug (DMD), many medication have been available for the treatment of MS, with different mechanisms of action, and different effectiveness and safety profiles [69].

Therefore, corticosteroids and DMDs are currently used for the treatment of multiple sclerosis. Corticosteroids are indicated for the treatment of acute and disabling relapses such as optic neuritis, significant motor disability, or acute ataxia, and even if the exact mechanism of action is not clear, antiedema effects, stabilization of the blood–brain barrier, reduction in proinflammatory cytokines, and apoptosis of T cells may be potential mechanisms of disease control [70].

The disease-modifying drugs are used for the relapsing forms of MS and have multiple benefits such as decreases in the frequency and severity of relapses and lower risk of development of disability with finally improvement in quality of life.

In patients with treated multiple sclerosis, several different side effects are attributed to corticosteroids and DMD use, including the development of drug-induced liver injury (DILI), defined as hepatocellular injury caused by medication or other xenobiotics leading to abnormalities in liver function, or triggering a self-perpetuating autoimmune process.

Generally, the 3 different clinical scenarios may be observed in patients taking medications: 1) *AIH with DILI* in patients with pre-existing and unknown AIH; 2) *drug-induced AIH*, when AIH is triggered by drugs; 3) *autoimmune-like DILI* when liver injury has features similar to that of autoimmune hepatitis.

Individuals with autoimmune-like DILI typically have a rapid response after cessation of the causative drug with or without immunosuppression, and, however, when immunosuppressive agents are given, the duration of treatment is relatively short [71].

In contrast, the differentiation between AIH and drug-induced AIH still remains a challenge because clinical and histological features are very similar or identical. However, only 2%-9% of cases of AIH are considered to be induced by drugs [72].

The Simplified Scoring System of the International Autoimmune Hepatitis Group which includes weighted scores for individual serological, genetic, and liver histological features is considered a useful tool for the identification of AIH and differentiation from drug-induced AIH. The strength of association between drug exposure and clinical manifestations (causality assessment) should always be considered in the diagnostic flow-chart.

#### Disease-modifying therapies

As for untreated MS, data on the relationship between treated MS and autoimmune hepatitis are limited because very few case reports are discussed in the literature.

Table 2 summarizes the available publications describing cases with coexistence of treated MS and AIH.

**Table 2:** AIH in treated MS

Author	Country	Number of patients	Age (years)	Gender	Autoantibodies	Liver biopsy	MS treatment	Previous MS treatment	AIH treatment
Kowalec et al. [73]	Canada	1	42	F	ANA	No	IFNβ	–	–
Corrieri-baizeau et al. [74]	France	2	56 (patient 1) 43 (patient 2)	F (patient 1) M (patient2)	ANA	Yes	Not reported	–	Corticosteroids (patient 1) Corticosteroids + azathioprine (patient 2)
Ferrò et al. [75]	Italy	1	19	F	ASMA	Yes	MP	–	MP Prednisone
Rigopoulou et al. [64]	Greece	13	n.a	n.a	n.a	Yes	Steroids ± IFNβ	–	Azathioprine Mycophenolate Mofetil Prednisolone
Nociti et al. [76]	Italy	3	24 (patient 1) 19 (patient 2) 59 (patient 3)	F	ANA + ASMA (patient 1) ASMA (patient 2) Negative (patient 3)	Yes	MP	–	Azathioprine Budesonide UDCA
Oliveira et al. [77]	Portugal	1	33	F	ANA	yes	MP Cyclophosphamide Glatiramer	–	MP
Farkas et al. [65]	US	1	40	F	ANA	Yes	Glatiramer IFNβ	–	Steroids Azathioprine
Kimura et al. [78]	Japan	1	49	F	negative	Yes	MP		–
Villamil et al. [79]	Argentina	2	20 (patient 1) 47 (patient 2)	F	ANA + ASMA (patient 1) ANA (patient 2)	yes	MP + IFNβ (patient 1) IFNβ (patient 2)	–	Prednisone azathioprine
Sayin [80]	Turkey	3	36 (patient 1) 38 (patient 2) 45 (patient 3)	F	Negative	Yes	IFNβ	–	Steroids Azathioprine
Yamaguchi [81]	Japan	1	44	M	Negative	yeS	MP + IFNβ		Prednisolone
Pulicken et al. [82]	US	1	43	F	ANA ASMA	Yes	IFNβ	–	MP Prednisone Azathioprine Mycophenolate Mofetil
El Sankari et al. [83]	Belgium	1	25	F	ANA ASMA ANCA	Yes	Alemtuzumab	–	Prednisolone Azathioprine
Martinez-Lapiscina et al. [84]	Spain	1	51	F	ASMA	Yes	Natalizumab	IFNβ	MP
Carlson et al. [85]	US	1	33	F	Not reported	Yes	Alemtuzumab	Fingolimod	Prednisone
Bolte et al. [86]	Germany	1	36	F	ANA ASMA	Yes	Alemtuzumab	interferon, natalizumab, glatiramer dimethyl fumarate	Prednisone Mycophenolate Mofetil Cyclosporine A
Canham et al. [87]	UK	1	43	F	Anti-LKM1	Yes	Alemtuzumab	–	Prednisolone Azathioprine
Arruti et al. [88]	Spain	1	n.a	n.a	n.a	n.a	glatiramer	n.a	n.a
Takahashi et al. [89]	Japan	1	43	F	negative	Yes	MP + IFNβ	–	Prednisolone UDCA
Neumann et al. [90]	Germany	1	71	M	ANA	Yes	glatiramer	IFNβ	Budesonide Mycophenolate Mofetil
Lisotti et al. [91]	Italy	1	31	F	ANA	No	natalizumab	IFNβ	MP Azathioprine
Kalafateli et al. [92]	Greece	1	57	F	ANA ASMA Anti-LKM3 Anti-SLA/LP	Yes	IFNβ	–	Prednisolone Azathioprine
Holmøy et al. [93]	Eudra Vigilance	1	n.a	F	n.a	n.a	n.a	n.a	n.a
Antezana et al. [94]	US	1	26	F	ASMA	Yes	Natalizumab	Steroids	–

*MP* methylprednisolone; *n.a.* not available

The largest population was described by *Rigopoulou *et al. who observed between 2005 and 2017 184 patients with MS and AIH was diagnosed in 14 patients. Thirteen out of 14 patients were under treatment: 5 patients with IFNβ plus methyl-prednisolone pulses, 3 with IFNβ plus oral steroids, 1 with IFN, 4 with methylprednisolone pulses [64]. AIH occurred between 1 and 120 months after starting IFNβ therapy (median time 12 months). Eleven out of 14 patients showed a significant increase in alanine transaminase (ALT) levels (> 10 upper limit of normal-ULN), while the remaining patients had ALT levels > 5 ULN. One patient developed jaundice; however, no cases of acute liver failure were reported. All patients had detectable autoantibodies and liver biopsy showed histological features consistent with AIH (interface hepatitis, plasma cell-rich lymphoplasmacytic infiltrates, hepatocellular rosette and emperipolesis) in 11 out of 13 patients, whereas three patients had minimal and unspecified changes of liver histology.

All patients were treated with azathioprine or mycophenolate mofetil and prednisolone with a good response to treatment [64].

Among selected papers, interferon beta is frequently reported as a treatment regimen for MS treatment. Type I interferons, including interferonα (IFNα) and interferonβ (IFNβ), are recognized as key cytokines involved in the host response to viral infection. However, this protective role is counterbalanced by potential for this cytokines to promote immune system activation and autoimmunity. The opposing roles in immunity, and therefore, their effects are beneficial or detrimental depending on different activated pathways and whether IFN pathway activation is transient or sustained over time [95].

The exact mechanisms of action of interferons are complex and not fully understood; however, it is clear that interferonβ has immunomodulatory and antiproliferative properties such as stimulation of the antinflammatory IL-10 release, down regulation of histocompatibility complex (MHC) class II expression present on the antigen-presenting cells, inhibition of T-cell migration through the blood–brain barrier, suppression of T cell activation and differentiation of neural stem cells to oligodendrocytes [96].

Nevertheless, the occurrence or recurrence of autoimmune diseases is a well-known side effect of type I IFN therapy. Long-term experience in chronic viral hepatitis and lymphoproliferative disease treatment has shown the occurrence of autoimmune disorders, such as thyroiditis, hepatitis, or diabetes during IFNα therapy.

Autoimmune events such as autoantibody development and thyroid dysfunctions, have been reported in patients with MS treated with interferonβ. About 37% of MS patients treated with interferon show alteration of liver function, whereas 20% of patients develop autoantibodies [97].

Several authors reported cases of AIH (Table 2) after IFN treatment, and almost all patients had positive autoantibodies (type 1 AIH).

We found six cases of AIH in patients receiving alemtuzumab or natalizumab. Both of them are neutralizing humanized monoclonal antibodies. Natalizumab inhibits the migration of leukocytes into the central nervous system by blocking the leukocyte α4 integrin and, finally, leukocyte adhesion to the vascular cell adhesion molecule 1 receptor on endothelial cells.

Alemtuzumab selectively targets CD52, an antigen highly expressed on T and B lymphocytes with depletion of circulating T and B cells shifting cytokine production toward a less inflammatory pattern [98].

Four cases of AIH after alemtuzumab treatment have been reported in the literature (Table 2). Another case has been described by *Holmøy *et al., who reported an analysis from EudraVigilance database a fatal autoimmune hepatitis probably alemtuzumab-related [93].

Mechanisms of liver injury are unknown, however, *Baker *et al. have suggested that a more rapid CD19 + B-cell reconstitution post-alemtuzumab therapy may be involved in secondary autoimmunity. Indeed, alemtuzumab depletes CD4 + T cells by more than 95%, including regulatory cells (-80%) and CD8 + T cells (> 80% depletion). CD19 + B cells are initially also depleted (> 85%), but after alemtuzumab administration, marked hyper-repopulation of immature B cells with conversion to mature B cells may occur. In the absence of effective T-cell regulation (via CD4 T regulatory cells and CD8 regulatory/suppressor cells), the escape of autoreactive B cells may be associated with a rapid development of alemtuzumab-neutralizing antibodies and subsequent occurrence of secondary B-cell autoimmunity [99].

Three cases of autoimmune hepatitis after natalizumab use have been reported in the literature (Table 2).

Additional cases have been reported, however, a definite diagnosis has not been established because features of drug-induced lived disease and autoimmune hepatitis coexisted (autoantibodies, histological pattern of plasma cell infiltration, no recurrence after steroid withdrawal) [98, 100].

We found only two cases of AIH after glatiramer acetate administration. Glatiramer acetate is a synthetic amino acid polymer similar to myelin basic protein and used for treating of relapsing forms of MS. Different potential mechanisms of action have been proposed, such as Th2 deviation of T cells, restoration of frequency and function of T regulatory cells, and immunomodulatory effects on antigen presenting cells.

Immunomodulation may induce the release of cytokines like IL-4, IL-6, and IL-10, which may enhance the production of autoantibodies and lead to autoimmunity in genetically predisposed patients [90].

#### Steroids

Finally, we found 5 cases of AIH in patients treated with steroids. The occurrence of autoimmune disease following administration of methyl prednisone (MP) seems to be illogical because intravenous corticosteroid treatment is one of the most efficient therapeutic options for severe exacerbations of many autoimmune diseases.

However, current literature shows that corticosteroids are not entirely safe for the liver.

The mechanisms of corticosteroid-induced liver injury are unclear and are only occasionally are related to the reactivation of occult HBV infection or to the excipient of the steroid preparation.

Some authors suggested that autoimmune hepatitis can be a consequence of an immune rebound phenomenon after steroid administration [101] and that steroid treatment may unmask AIH in predisposed patients [102].

*Nociti *et al. observed one hundred and seventy-five patients treated with pulsed methylprednisolone therapy for a clinical or neuroradiological relapse. All patients received i.v. methylprednisolone at the dosage of 1,000 mg/day for 5 days. The authors collected data on 251 cycles of i.v. steroid treatment in 175 patients with MS. 171 patients had normal ALT levels at baseline. Two weeks after the steroid treatment, serum ALT elevation (any grade) was observed in 8.6% of cycles. Six patients experienced a severe liver injury and underwent liver biopsy. Three of them had histological features consistent with autoimmune hepatitis. Two patients had detectable autoantibodies (ANA + ASMA and ASMA, respectively). Two patients received immunosuppressive treatment (azathioprine + budesonide) with complete normalization of liver function test 6 months later [76].

*Zoubek *et al*.* performed a revision of the literature and found 50 published cases of MP hepatotoxicity. Eighty-six percent of the patients were female with MS or Graves' ophthalmopathy. Cases showed a typical onset of hepatocellular injury 6 weeks after starting treatment. Four patients died, whereas the rechallenge occurred in 19 cases. The author suggested that, due to a potent immunosuppressive effect, the methylprednisolone may induce a strong but transient immunosuppression, followed by an immune reconstitution, which could finally awaken an autoimmune-like reaction in a susceptible host. Therefore, liver monitoring during and after high-dose methylprednisolone therapy should always be performed.

## Primary biliary cholangitis and multiple sclerosis

### Primary biliary cholangitis: pathogenesis, diagnosis and clinical presentation

Primary biliary cholangitis (PBC), formerly known as primary biliary cirrhosis, is a chronic inflammatory autoimmune cholestatic liver disease whose diagnosis is based on the presence of serum liver tests indicative of a cholestatic hepatitis in association with circulating antimitochondrial antibodies (AMA) or specific ANA reactivity (anti-sp100 and anti-gp210) and histologic evidence of chronic non-suppurative, granulomatous small bile duct cholangitis [103, 104]. The disease is progressive and may result in end-stage liver disease and its associated complications. The incidence is generally between 1 and 2 per 100,000 population per year and the disease is female predominant [104, 105].

In 2021, a study published by Gazda et al. have shown that the pooled point-prevalence rate of PBC in Europe between 2000 and 2020 was 22 cases per 100.000 inhabitants, whereas the pooled annual incidence rate is 1.87 new cases per 100.000 inhabitants [106]. The incidence and prevalence vary across regions and have an increasing tendency over time. Lv et al. recently showed that the annual incidence of PBC ranges from 0.23 to 5.31 per 100 000 persons, with the lowest reported in Estonia and the highest in Italy where the annual incidence in 2015 was three times higher than that reported in 2004–2009 [107].

The youngest patient with PBC reported in the literature was 15 years old, whereas no cases have been observed in pediatric patients [104, 108].

The etiology of PBC is thought to be due to a combination of genetic risk factors and environmental triggers, whereas the serologic hallmark is the AMA, which is specific for bile duct pathology [103].

Aberrant expression of MHC-II subregion genes (HLA-DP, HLA-DR, and HLA-DQ) can be detected on biliary epithelial cells in the early stages of PBC, whereas their expression decreases in advanced disease. In this context, it is noteworthy that the specific HLA-DR and HLA-DQ loci are associated with an increased risk or protection for PBC [109].

AMA targets the lipoic acid present on the 2-oxo-acid dehydrogenase complexes located on the inner mitochondrial membrane [110]. Xenobiotics and their metabolites (such as acetaminophen) may mimic or modify lipoic acid and lead to the loss of tolerance [111].

Moreover, several case–control studies have found an association between PBC and urinary tract infections by Escherichia coli because human PDC-E2 is molecularly similar to E. coli PDC-E2 [112].

The unsolved questions are why ubiquitous autoantigens have been linked to specific targeting of the biliary epithelial cells in the PBC pathogenesis and how autoantigens located on the inner mitochondrial membrane may be involved in AMA production [103].

AMA is immunoglobulin A (IgA) that undergoes transcytosis through the biliary cells and causes mitochondrial dysfunction. The immune attack may be consequent to the incomplete proteolysis of the pyruvate dehydrogenase complex PDC-E2 or other mitochondrial proteins during apoptosis of biliary cells, which is a hallmark of this cell type [113, 114].

It is estimated that 0.6% of the general population has AMA, whereas only 16% of patients with AMA develop PBC [115, 116]. In some populations, the prevalence may be higher, such as people of native Cheyenne origin; in this group, the prevalence of AMA or ANA specific for PBC is 15% even in the absence of clinical evidence for PBC [117].

AMA is found in 95% of PBC patients, whereas antinuclear antibody and antismooth muscle antibody are found in nearly 50% [118]. Antinuclear antibodies, including sp100 and gp210, can be detected in more than 30% of AMA-negative PBC patients [103, 104].

PBC is a chronic cholestatic disease, therefore, its clinical features include fatigue, pruritis, jaundice, xanthomas, osteoporosis, and dyslipidemia. However, up to 60% of PBC patients are asymptomatic at diagnosis, and the median time from diagnosis to the appearance of symptoms ranges between 2 and 4.2 years [103, 119]. Current guidelines recommend that in adult patients with cholestasis, in the absence of systemic diseases, a diagnosis of PBC can be made based on elevated ALP and the presence of AMA at a titer > 1:40. The diagnosis does not require liver biopsy unless PBC-specific antibodies are absent. In the absence of effective treatment, the median time to develop significant liver fibrosis is 2 years, whereas 25% of untreated patients develop liver failure by 10 years after diagnosis [104, 120].

### Genetic risk factors for primary biliary cholangitis and multiple sclerosis

Human leukocyte antigen (HLA) class II alleles have been associated with PBC onset; however, PBC patients may also have genetic risk factors in non-HLA regions [121].

Several studies have provided evidence that PBC is associated with DRB1*08 as a predisposing allele and DRB1*11 and DRB1*13 as protective alleles [122, 123]. Moreover, additional studies recently found that HLA DR*07 and *08 alleles may be risk factors for PBC in some populations, while DR*11, *12, *13, and *15 alleles may play a protective role [124].

GWASs aiming to study genetic susceptibility in PBC patients confirmed that the HLA class II domain has the strongest association with PBC susceptibility and indicated the involvement of HLA class II polymorphisms in the pathogenesis of PBC [121]. However, HLA alone does not explain the genetic predisposition to PBC because about 80% of PBC patients do not carry the most common HLA susceptibility genotypes; therefore, other genes are probably involved in the disease development. GWASs have also identified 44 non-HLA PBC predisposition loci [121] A significant association has been shown between PBC and genetic variants of *interleukin* (*IL*)*12A*, *IL12RB2*, *interferon regulatory factor 5* (*IRF5*), *transportin 3* (*TNPO3*), and, finally, *transcription factor Spi*-*B* (*SPIB*) encoding a transcription factor involved in B-cell receptor and T-cell signaling [125]. The IL-12 signaling pathway appears to be a key player in the pathogenesis of PBC worldwide [113], and both animal models of PBC and pediatric cases of congenital IL-12 deficiency have been associated with the development of PBC [126].

Genetic overlap between common autoimmune diseases has been largely studied; however, few data are available for MS and PBC.

*Olafsson *et al*.* studied polygenic risk scores based on public summary statistics of variants outside the major histocompatibility complex region to quantify genetic overlap between common autoimmune diseases in Icelanders and identify disease clusters. They found that for patients having double risk of PBC, MS risk increased by 29%, whereas polygenic risk scores corresponding to double risk of MS increased the risk of PBC by 81% [127].

### Primary biliary cholangitis in patients with multiple sclerosis: literature data

The co-existence of MS and PBC has been rarely reported in literature. *Sattar *et al*.* reported 3 cases of MS and PBC in female patients; 2 out of them had PBC before MS diagnosis [14].

Only a few studies have reported the prevalence of PBC in patients with multiple sclerosis.

*Nielsen et* al. used 3 national registries to estimate the RRs for 42 different autoimmune diseases in a population-based cohort of 12 403 MS patients. Only one patient with PBC has been reported [22].

Five studies reported the prevalence of PBC in MS patients from Europe, Australia, and US.

The prevalence of primary biliary cirrhosis was very low, ranging from 0–0.12%.

Three of five studies compared the prevalence of PBC in MS patients versus the control group and no differences were observed. In particular, *Seyfert *et al*.* screened 101 MS patients and no cases of PBC were observed [128]. Similar results were reported by *Henderson *et al*.* who observed 117 patients with MS but no patients developed PBC. Finally, *Langer-Gould *et al*.* studied a large MS population (*N* = 5296) and only 1 patient was diagnosed with PBC before the diagnosis of MS (0.02%) versus 5 cases out of 26,478 MS patients in the control group (0.02%) [129].

*Finally, Tsuris et* al. studied the frequency of PBC-specific autoantibodies in a cohort of 133 MS patients. AMA-M2 were detected in 1.5% of patients, whereas anti-sp100 and anti-gp210 were detected in less than 1% of the study population. Two patients were diagnosed with PBC [62].

## Primary sclerosing cholangitis and multiple sclerosis

### Primary sclerosing cholangitis: pathogenesis, diagnosis and clinical presentation

Primary sclerosing cholangitis (PSC) is a chronic liver disease characterized by inflammation and leading to multifocal biliary fibrotic strictures and cirrhosis [130, 131].

The etiology of PSC is unclear; however, immune responses against self-antigens expressed by biliary cells have been proposed to play a pivotal role in the pathogenesis [132].

The etiology and pathogenesis of PSC are not well understood, and several hypotheses related to the etiology of PSC have been proposed over time.

It has been hypothesized that abnormalities in the complex relationship between the gut and hepatobiliary system could promote the development of PSC. This model may explain the link between PSC and inflammatory bowel disease. Three potential key pathogenic mechanisms may play a role in the interplay of PSC and IBD: altered gut microflora (“intestinal dysbiosis”) that produces potentially toxic or immunostimulatory molecules, increase intestinal permeability due to enteric inflammation and subsequent translocation of microbial toxins and bacteria, and immune activation and stimulation triggered by intestinal bacteria, resulting in biliary injury and fibrosis [133, 134].

A different theory supports the hypothesis that lymphocytes activated in the bowel of IBD patients could be aberrantly recruited to extraintestinal sites such as the liver. A network of adhesion molecules and chemokine receptors could be expressed in the liver, leading to the recruitment of intestinal lymphocytes through the enterohepatic circulation [135, 136]. The mechanisms involved in the aberrant expression of adhesion molecules and chemokines in the liver remain unknown.

A third theory by Fickert et al. hypothesizes a role for bile acid toxicity due to impaired secretion of phospholipids and subsequent cholesterol-supersaturated bile with an oxidative process similar to the process of atherosclerosis, necrosis, and apoptosis [137].

The inflammatory infiltrate of PSC is largely comprised of T cells; however, NK cells, macrophages, and B cells may be involved in the immunopathogenesis of PSC. Pathogen-associated molecular patterns (PAMPs) such as lipopolysaccharide (LPS), lipoteichoic acid, peptidoglycans, and unmethylated bacterial dinucleotide motifs could enter the portal circulation through an inflamed and, therefore, permeable intestine. PAMPs could activate macrophages, dendritic cells, and NK cells through pattern recognition receptors, including Toll-like receptors (TLRs) leading to the secretion of cytokines which in turn activate NK cells (IL-12) and promote recruitment and activation of lymphocytes (TNF-α, IL-1β, and CXCL8) [134].

The association with inflammatory bowel disease (IBD) is a hallmark of this disease because IBD is reported in most patients. The typical PSC patient is a 30–to-40-year old male presenting with a diagnosis of ulcerative colitis or Crohn’s colitis. In these patients, the increased risk of biliary cancer and colorectal cancer in PSC is widely established and of major clinical relevance with a prevalence of about 10% [130, 131].

Even if epidemiological studies have reported a prevalence rate of 1 per 10,000 and an incidence rate in Europe and the US between 0.4 and 2.0 per 100,000 per year, several studies have shown that the incidence of PSC is increasing [17, 138].

Trivedi et al. performed a systematic review of population-based studies in 2022 to quantify the global epidemiology of primary sclerosing cholangitis. Different results were observed across countries, however, studies from Norway, The Netherlands, US, and UK showed a significant increase over time between 2000 and 2007, whereas, the age of onset was between 40 and 59 years [139].

In PSC patients, several autoantibodies have been observed, which react with biliary or colonic epithelial and cytoplasmic antigens of neutrophil granulocytes [132]. According to the current guidelines, autoantibodies should not be used for the diagnosis of PSC because they are intermittently positive, and therefore, they have wide ranges of frequencies in literature [140, 141].

Anti-nuclear antibodies have been reported in 8–77% of patients, smooth muscle antibody in up to 83% and the anti-neutrophil cytoplasmic antibody (ANCA) in 26–96% [142]. ANCA has different staining patterns on immunofluorescence: cytoplasmic (c-ANCA), targeting the cytoplasmic protein leukocyte proteinase 3 (PR3-ANCA), perinuclear (p-ANCA), targeting another cytoplasmic protein, myeloperoxidase, atypical p-ANCA (perinuclear anti-neutrophil nuclear antibody), directed against components of the nuclear envelope. People with PSC show p-ANCA and atypical p-ANCA.

PSC requires a radiological diagnosis, and MR Cholangiography is the best modality for the diagnosis of PSC with good sensitivity, specificity (0.86 and 0.94, respectively), and cost-effectiveness compared with endoscopic retrograde cholangiography (ERC) [140]. Serum liver tests typically show cholestasis, but it is important to know that alkaline phosphatase (ALP) levels can fluctuate naturally in PSC and may be normal in a significant percentage of patients [131, 143].

The genetic risk of PSC was studied in the largest genetic study (*N* = 3789 cases), which assays thousands of SNPs of significance from previous studies in other immune-mediated diseases and found multiple risk loci for PSC [144].

### Genetic risk factors for primary sclerosing cholangitis and multiple sclerosis

The cause of PSC remains is unknown; however, association with HLA genotypes has been recognized in PSC [145].

Moreover, a subset of genes involved in bile homeostasis and mutations in the gene encoding the cystic fibrosis transmembrane receptor and recurrent bacterial infections may be key components of the genetic architecture of PSC [146].

BACH2 and IL2RA, which are considered susceptibility loci for primary sclerosing cholangitis, have been previously reported as genetic risk factors for multiple sclerosis [130].

The BACH2 is a transcription factor predominantly expressed in B and T lymphocytes and acts as a repressor that controls the terminal differentiation and maturation of both B and T lymphocytes [147].

Only 1 case describing concurrent MS and PSC in a 41-year-old men has been reported in 2003 [148].

Because of elevated liver enzymes, the patients underwent endoscopic retrograde cholangiopancreatography (ERCP), which showed evidence of PSC, whereas liver biopsy showed stage 2 fibrosis. He was started on ursodeoxycholic acid with a subsequent improvement in liver function tests.

Due to the lack of additional cases in the literature, the association between MS and PSC could have been by chance. However, the well-known association between IBD and MS on one hand, and PSC and IBD on the other, suggests that a potential link between MS and PSC should be considered.

## Conclusion

The occurrence of abnormal liver enzyme levels in patients with multiple sclerosis should always be investigated, and autoimmune liver diseases should be undoubtedly considered in the differential diagnosis. The inclusion of hepatologists in the multidisciplinary care unit can optimize the management of multiple sclerosis and, at once, allow for the early diagnosis of autoimmune liver disease.

## Abbreviations

MS: Multiple sclerosis
AIH: Autoimmune hepatitis
PSC: Primary sclerosing cholangitis
PBC: Primary biliary cholangitis
ALP: Alkaline phosphatase
ANA: Antinuclear antibody
ASMA: Anti-smooth muscle antibody
anti-LKM1: Anti-liver/kidney microsomal antibody type 1
anti-LKM3: Anti-liver/kidney microsomal antibody type 3
anti-LC1: Antibodies against liver cytosol type 1 antigen
ERCP: Endoscopic retrograde cholangiopancreatography
DC: Dendritic cell
APC: Antigen-presenting cell
IBD: Inflammatory bowel disease
SNP: Single-nucleotide polymorphism
Anti-SLA: Anti-soluble liver antigen
pANCA: Perinuclear antineutrophil cytoplasmic antibodies
AMA: Antimitochondrial antibody
UC: Ulcerative colitis
DMD: Disease-modifying drug
DMT: Disease-modifying therapy
IFNα: Interferonα
IFNβ: Interferonβ
IL2RA: Interleukin 2 receptor subunit alpha
CD25: Cluster of differentiation 25
CD122: Cluster of differentiation 122
IL2: Interleukin 2
Treg cells: Regulatory T cells
DILI: Drug-induced liver injury
BACH2: BTB Domain And CNC Homolog 2
